# Circulatory microRNA signature distinguishing rheumatoid arthritis and psoriatic arthritis

**DOI:** 10.1093/rheumatology/keag246

**Published:** 2026-05-08

**Authors:** Órla Tynan, Megan M Hanlon, Achilleas Floudas, Siobhán Wade, Ursula Fearon, Douglas J Veale

**Affiliations:** Molecular Rheumatology, Trinity Biomedical Sciences Institute, Trinity College Dublin, Dublin, Ireland; EULAR Centre of Excellence, Centre for Arthritis and Rheumatic Diseases, St Vincent’s University Hospital, Dublin, Ireland; Molecular Rheumatology, Trinity Biomedical Sciences Institute, Trinity College Dublin, Dublin, Ireland; EULAR Centre of Excellence, Centre for Arthritis and Rheumatic Diseases, St Vincent’s University Hospital, Dublin, Ireland; Division of Rheumatology, Inflammation, and Immunity, Brigham and Women’s Hospital and Harvard Medical School, Boston, MA, USA; Molecular Rheumatology, Trinity Biomedical Sciences Institute, Trinity College Dublin, Dublin, Ireland; EULAR Centre of Excellence, Centre for Arthritis and Rheumatic Diseases, St Vincent’s University Hospital, Dublin, Ireland; Molecular Rheumatology, Trinity Biomedical Sciences Institute, Trinity College Dublin, Dublin, Ireland; Molecular Rheumatology, Trinity Biomedical Sciences Institute, Trinity College Dublin, Dublin, Ireland; EULAR Centre of Excellence, Centre for Arthritis and Rheumatic Diseases, St Vincent’s University Hospital, Dublin, Ireland; EULAR Centre of Excellence, Centre for Arthritis and Rheumatic Diseases, St Vincent’s University Hospital, Dublin, Ireland

**Keywords:** autoimmune disease, rheumatoid arthritis, psoriatic arthritis, miRNA, biomarkers

## Abstract

**Objectives:**

Gene regulatory microRNAs (miRNAs) have emerged as promising biomarkers and therapeutic targets in autoimmunity pathology. This study examines circulatory miRNAs as cellular biomarkers that can distinguish rheumatoid arthritis (RA) from psoriatic arthritis (PsA) to evaluate the potential implications for disease pathogenesis.

**Methods:**

RA (*n* = 48) and PsA (*n* = 49) patients and healthy controls (HC) (*n* = 20) were recruited and serums obtained. Multiplex analysis of serum miRNAs was performed using the FirePlex miRNA Immunology-V2 panel (FirePlex Bioworks Inc.). DNA intelligent analysis (DIANA)-mirPath and STRING software were used to predict pathways targeted by the dysregulated miRNAs.

**Results:**

Seven miRNAs, miR-126-3p, miR-29b-3p, miR-22-3p, miR-223-3p, miR-320a, let-7e-5p and let-7g-5p, were significantly elevated in RA serum compared with PsA (all *P* < 0.05), in addition to HC (all *P* < 0.05), with high sensitivity and specificity as determined by receiver operating characteristic curve analysis. Principal component analysis and biplot analysis demonstrated differential miRNA clustering between both disease states with a dominant skew towards three specific miRNAs in RA *vs* PsA: miR-29b-3p, miR-22-3p and miR-223-3p. DIANA analysis and STRING visualization of this miRNA signature identified downstream target pathways including phosphoinositide 3-kinase–AKT and FoxO signalling, all importantly associated with aspects of RA pathogenesis including angiogenesis, invasion and cell death.

**Conclusion:**

This study identified three key miRNAs demonstrating differential expression levels between RA and PsA, which potentially govern downstream inflammatory pathways regulating distinct disease mechanisms. Therefore, circulating miRNAs may be valuable as non-invasive diagnostic biomarkers that can distinguish RA from PsA and may additionally assist in elucidating differential disease pathogenesis.

Rheumatology key messages

- Circulating serum miRNAs offer a minimally invasive, reliable method to distinguish RA from PsA.
- Circulating miRNAs assist in identifying differences in disease pathways, improving understanding of RA and PsA pathogenesis.
- Targeting specific miRNAs could reduce multiple mechanisms of synovial joint inflammation and tissue damage simultaneously.

## Introduction

MicroRNAs (miRNAs) are non-coding RNAs that mediate post-transcriptional gene regulation, primarily though induction of mRNA degradation and/or translational repression, effectively silencing gene expression [1]. Numerous studies highlight their central role in inflammatory pathologies, where aberrant miRNA activity governs both local and systemic inflammation through regulation of pro/anti-inflammatory genes, in addition to their ability to activate several additional signalling cascades, thus highlighting the scope of their involvement in inflammatory shifts [2].

In rheumatic diseases including rheumatoid arthritis (RA) and psoriatic arthritis (PsA), miRNA dysregulation is a key contributor to bone and cartilage destruction, in addition to the phenomenon of angiogenesis [3–5]. Stanczyk *et al*. [6] first identified miR-155 and miR-146a as being significantly elevated in RA synovial fluid compared with osteoarthritic (OA) controls, prompting extensive research into miRNA involvement in synovial inflammation and angiogenesis [3]. Indeed, in RA, miRNAs have been implicated in NLRP3 inflammasome activation in macrophages, the promotion of B cell responses, skewing of CD4^+^ T cell balance toward inflammation, and driving monocyte and dendritic cell differentiation into osteoclasts [7–10]. Consequently, miRNA dysregulation enhances TNF-α, IL-6, IL-1β, IL-8, IL-17A, MCP-1, RANTES and matrix metalloproteinase production, perpetuating joint destruction and synovial pathology [3, 11]. Moreover, although less defined, PsA also exhibits differential miRNA expression, including altered serum levels of miR-146a-5p, miR-21-5p, miR-130a-3p, miR-221-3p, miR-151-5p and miR-26a-5p, alongside synovial cell dysregulation of miR-941 and miR-23a [12–14].

Growing recognition of the central role of miRNAs in inflammatory disease has spurred investigation into their potential as biomarkers and therapeutic targets. Beyond cancer, where tissue and circulating miRNAs act as established oncogenic or tumour-suppressive markers in prostate, colorectal, lung, melanoma and breast cancers, miRNAs also serve as biomarkers in neurodegenerative disease, heart failure and sepsis [15–18]. In the context of autoimmune diseases, the identification of diagnostic miRNA signatures may allow earlier and more precise intervention [19]. Currently, RA diagnosis relies on rheumatoid factor, C-reactive protein, and multi-biomarker disease activity scores, with anti-citrullinated protein antibodies (ACPA) positivity as the hallmark biomarker [20]. However, ∼20% of RA patients are ACPA-negative, and not all ACPA-positive individuals develop disease, thus highlighting the need for additional biomarkers [21]. While single miRNAs may hold diagnostic value, for example miR-29a upregulation in acute pancreatitis [22], attention has shifted toward miRNA signatures—combinatorial patterns that improve diagnostic accuracy. For instance, in type-1 diabetes compared with healthy controls (HC), a distinct signature of nine upregulated miRNAs was detected, whilst similarly in chronic urticaria, a panel of five circulating upregulated miRNAs was found to be capable of distinguishing between the control and non-control cohorts [23, 24]. Distinct miRNA signatures have also been shown in inflammatory arthritis compared with healthy controls, levels that are perturbed pre-disease onset [13, 25].

Furthermore, the utility of miRNA signatures in distinguishing inflammatory pathotypes has been previously demonstrated in inflammatory bowel disease, where distinct circulating miRNA profiles differentiate ulcerative colitis from Crohn’s disease [26]. Moreover, Cheleschi *et al.* eloquently demonstrated a heightened level of circulating miR-140 in PsA compared with RA peripheral blood mononuclear cells (PBMCs) [27]. Additionally, Bonek *et al.* identified a specific profile of four miRNAs capable of distinguishing between RA, PsA, and ankylosing spondylitis while also correlating with disease activity and pro-atherogenic metabolism in the individual disease states [28]. However, despite this progress, minimal studies to date have directly compared circulating miRNA profiles between RA and PsA with this available research predominantly focusing on miRNA expression in PBMCs or smaller profiles of serum miRNA panels pre-selected based on functionality [27, 28]. Thus, this study focuses on differences in a significantly greater range of circulating miRNAs between RA and PsA cohorts and the downstream pathways they regulate, aiming to enhance understanding of disease-specific mechanisms and improve diagnostic precision.

## Methods and results

Ethics approval was granted by the St Vincent’s University Hospital Research Ethics Committee (Ref No: RS04-001) and informed consent was obtained.

### RA serum displays a distinct miRNA profile compared with PsA serum

**Figure 1:**
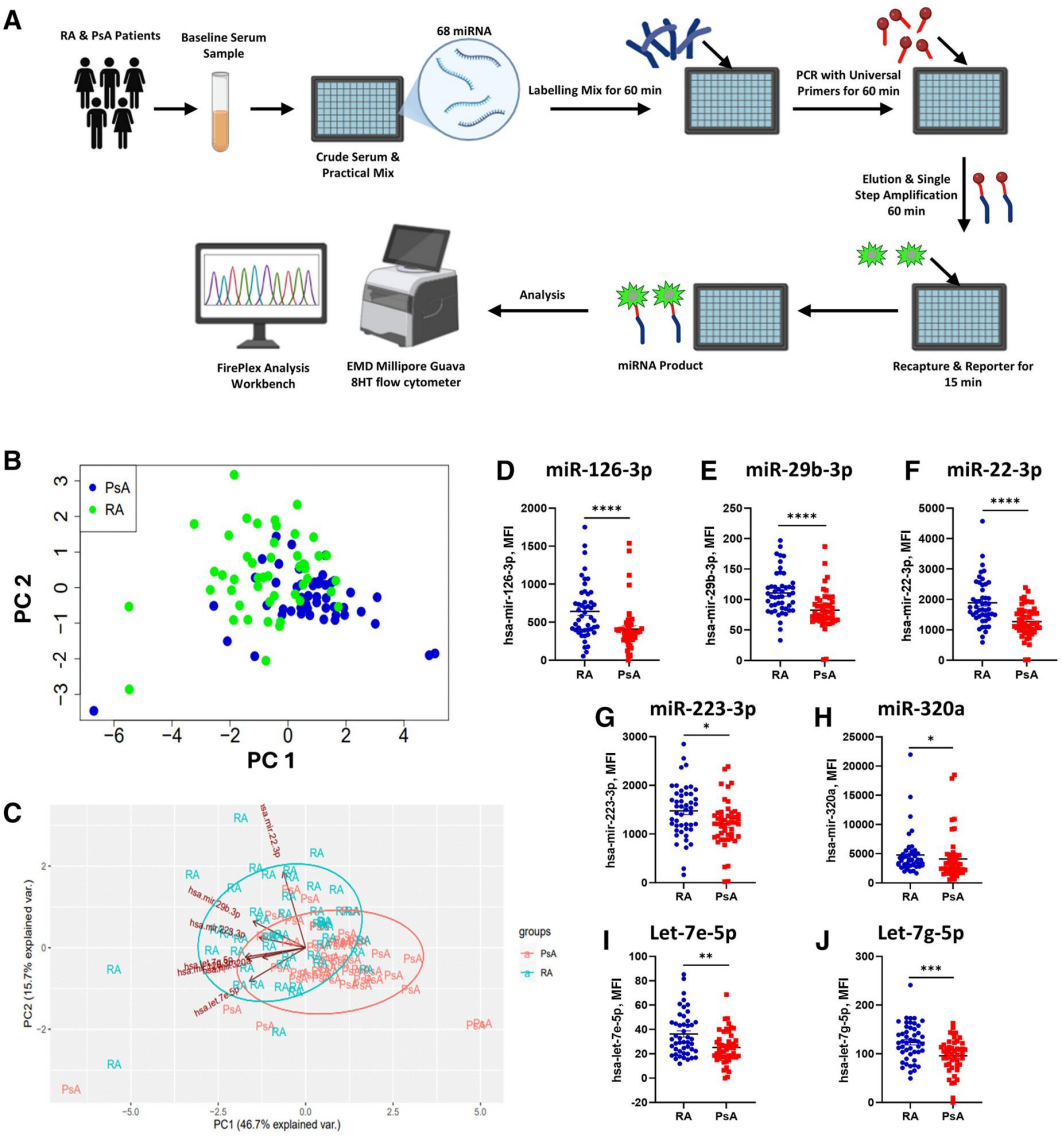
Analysis of microRNA (miRNA) expression for the top seven differentially expressed miRNAs between RA and PsA. (**A**) Process of RA and PsA patient serum miRNA analysis using the FirePlex miRNA assay (FirePlex Bioworks Inc.). (**B**) PCA analysis demonstrating serum miRNA expression between RA (*n* = 48) and PsA (*n* = 49). PCA analysis was performed in R (v4.0) function prcomp with scaling. (**C**) Biplot analysis of the seven significant miRNAs skewed towards RA *vs* PsA. Principal component analysis biplots were generated under R v4.0 with package ggbiplot v 0.5 with function ggbiplot. (**D–J**) Dot plot representations showing median fluorescence intensity (MFI) of the relative expression of the seven most differentially expressed serum miRNAs isolated from the serum of RA (*n* = 48) *vs* PsA (*n* = 49) cohorts and analysed and amplified using the Multiplex Circulating miRNA Assay. All data are represented as mean and SEM. Statistical analysis was performed using the non-parametric Mann–Whitney *U*-test with statistical significance defined by *P ≤ 0.05, **P ≤ 0.01, ***P ≤ 0.001, ****P ≤ 0.0001

**Figure 2:**
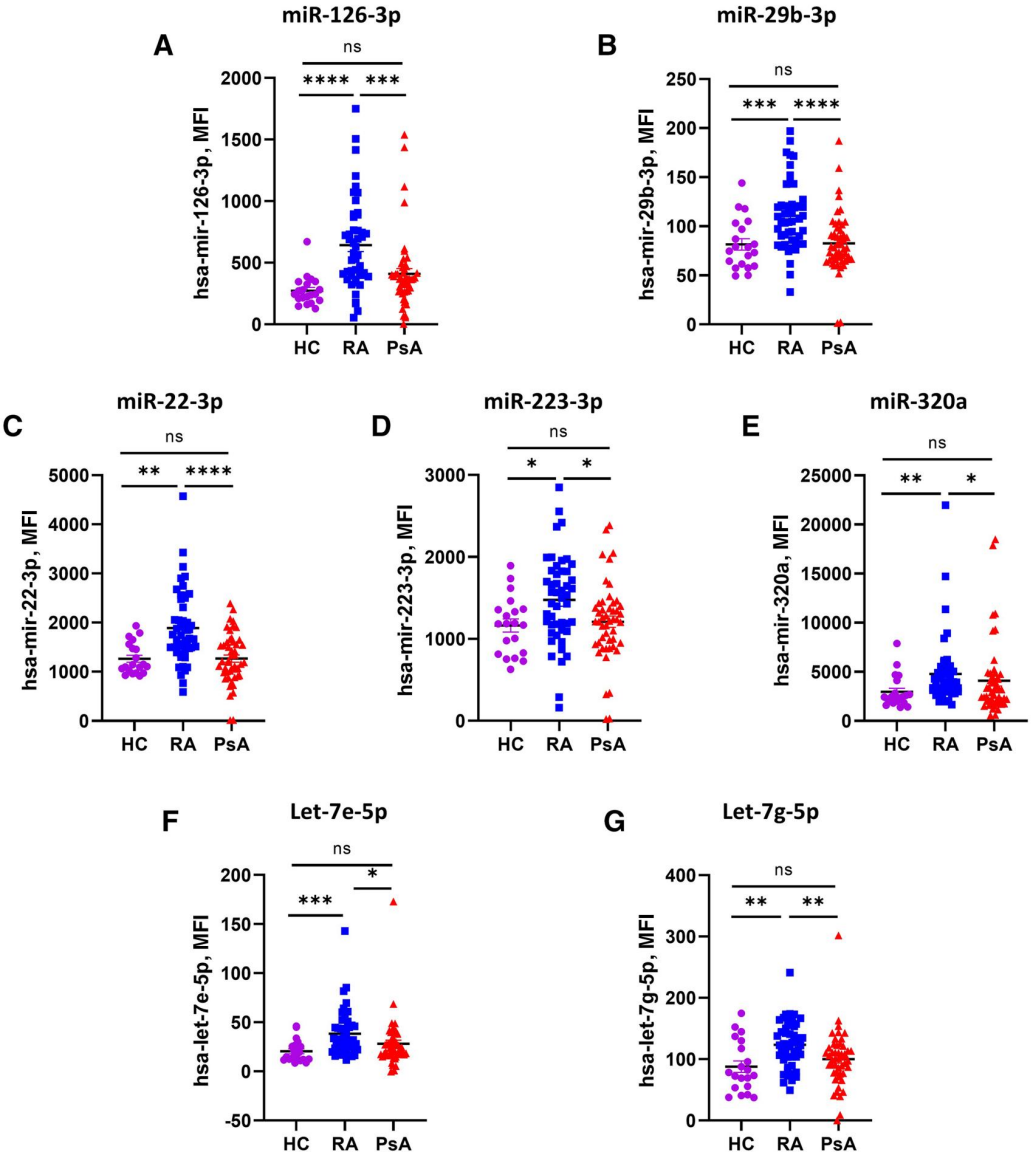
Expression of seven serum miRNAs that are significantly increased in RA compared with both PsA and healthy controls (HC). (**A–G**) Dot plot representations showing median fluorescence intensity (MFI) of the seven serum miRNAs of interest isolated from the serum of RA individuals (*n* = 48), PsA individuals (*n* = 49) and HC (*n* = 20) and analysed and amplified using the Multiplex Circulating miRNA Assay. All data is represented as mean and SEM. Statistical analysis was performed using the non-parametric, Kruskal–Wallis test with statistical significance defined by *P ≤ 0.05, **P ≤ 0.01, ***P ≤ 0.001, ****P ≤ 0.0001

In this study, serum was isolated from the blood of RA patients (*n* = 48) and PsA patients (*n* = 49), and the expression profile of a 68-miRNA immunology panel associated with immune dysregulation was determined using the Multiplex Circulating MiRNA Assay (Abcam, Cambridge, UK) (Fig. 1A). Clinical demographics are located in Supplementary Data S1. The FirePlex Analysis Workbench software (Fireplex Bioworks Inc., Abcam, Cambridge, UK) was used to analyse miRNA expression between RA and PsA. Significant differences in the expression levels of 19 miRNAs between RA and PsA cohorts were identified, with 16 miRNAs significantly higher in RA patients compared with the PsA group (all *P* ≤ 0.05) whilst conversely, three miRNAs, miR-203a-3p (*P* ≤ 0.001), miR-185-5p and miR-151-a-5p (both *P* ≤ 0.05), were significantly elevated in the PsA group (Supplementary Fig. S1A). A full list of the significantly different miRNAs is presented in Supplementary Table S1. Principle component analysis (PCA) of differentially expressed miRNAs demonstrated that specific RA patients clustered separately from PsA, although there is overlap with some patients between each group (Fig. 1B). Further interrogation using biplot analysis of the PCA plot identified that seven miRNAs were specifically skewed towards RA compared with PsA (Fig. 1C). Dot plots of these top seven differentially expressed miRNAs in RA compared with PsA are shown in Fig. 1D for miR-126-3p (*P* ≤ 0.0001), Fig. 1E for miR-29b-3p (*P* ≤ 0.0001), Fig. 1F for miR-22-3p (*P* ≤ 0.0001), Fig. 1G for miR-223-3p (*P* ≤ 0.05), Fig. 1H for miR-320a (*P* ≤ 0.05), Fig. 1I for let-7e-5p (*P* ≤ 0.01) and Fig. 1J for let-7g-5p (*P* ≤ 0.01). To establish their potential validity, we demonstrated that all seven identified miRNAs that were significantly increased in the RA patients *vs* the PsA were also significantly increased compared with the HC (Fig. 2A–G).

**Figure 3:**
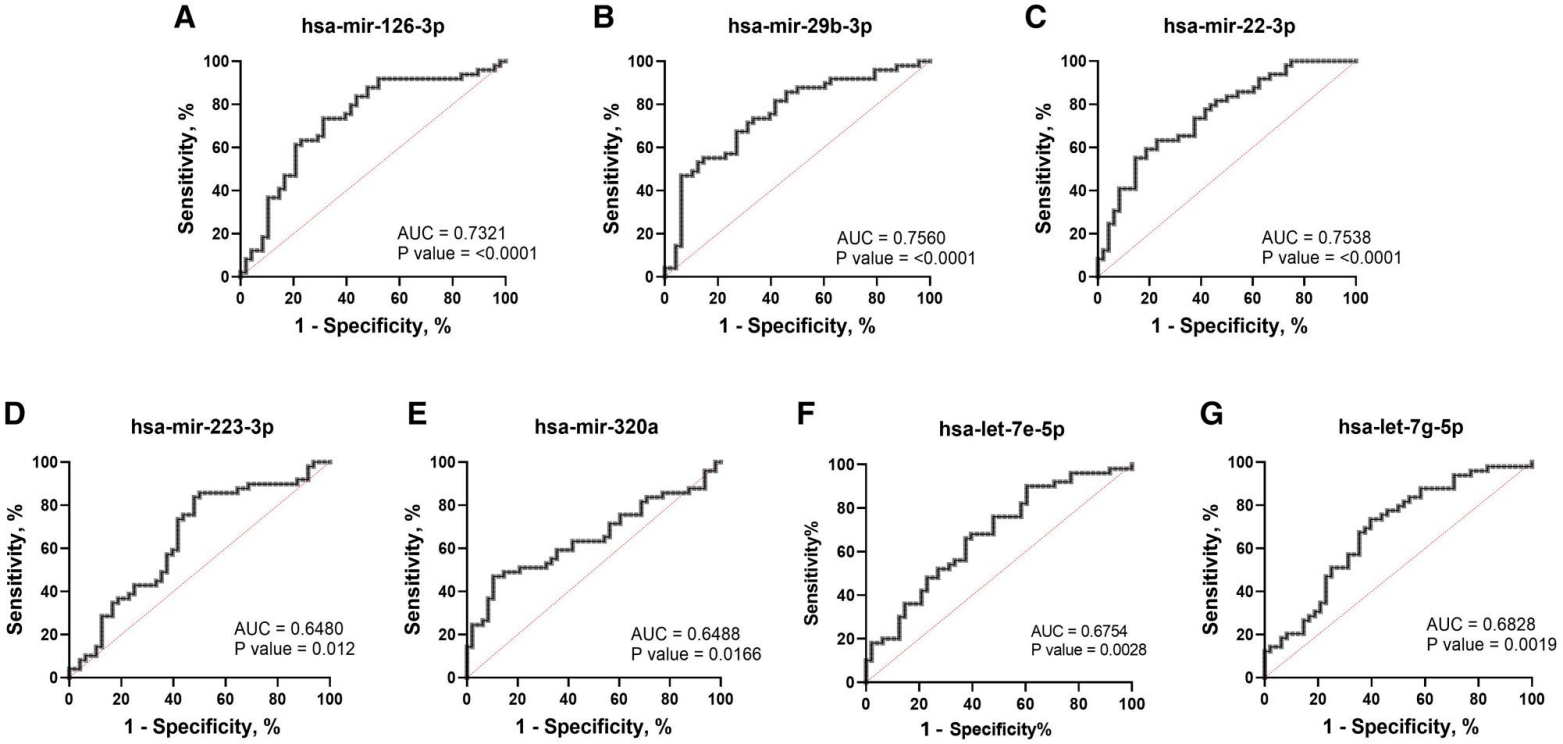
Receiver operating characteristic (ROC) curve analysis based on the expression levels of serum miRNA. (**A–G**) ROC curves of RA individuals (*n* = 48) based on the level of expression in serum of the seven miRNAs of interest compared with a PsA cohort (*n* = 49). The prediction accuracy with which each miRNA can differentiate between RA and PsA is represented by the area under the curve (AUC). AUC was determined with 95% confidence intervals

Furthermore, a receiver operating characteristic (ROC) curve analysis was performed to evaluate the sensitivity and specificity of each of the seven miRNAs to distinguish between RA and PsA patients (Fig. 3A–G). The strongest statistical separation was observed for miR-22-3p with an area under the curve (AUC) of 0.7538 (*P* < 0.0001) (Fig. 3C) followed by miR-126-3p and miR-29b-3p, with AUCs of 0.7321 (*P* < 0.0001) (Fig. 3A) and 0.7560 (*P* < 0.0001) (Fig. 3B), respectively. miR-223-3p, miR-320a, let-7e-5p and let-7g-5p (Fig. 3D–G) all demonstrated lower sensitivity and specificity with an AUC of <0.7. Interestingly, as shown in Supplementary Fig. S2, multivariate analysis for the strongest performing miRNA improved the predictive value slightly as compared with the individual miRNA, with the highest predictive value observed for miR-29b-3p, miR-22-3p and miR-320a with an AUC of 0.8151 (Supplementary Fig. S2D). Moreover, multivariate analysis for the complete serum miRNA signature generated an AUC of 0.7784, thus demonstrating a slightly higher prediction accuracy than each miRNA alone (Supplementary Fig. S2G).

### Effect of disease parameters, ACPA positivity and gender on the expression of the serum miRNA

No significant correlations were observed between the seven differentially expressed miRNAs and any clinical features in the RA or PsA patients indicating that these miRNAs are not associated with disease severity but rather the disease itself (Supplementary Figs S3 and S4). Furthermore, a sub-analysis demonstrated no significant differences for any of the miRNAs between ACPA^+^ (*n* = 11) *vs* ACPA^−^ (*n* = 18) patients indicating the biomarker value of these miRNAs in all RA patients irrespective of their ACPA status (Supplementary Fig. S5A–G).

Finally, to ascertain if gender may influence the expression of the miRNA associated with RA, patients in both disease states were stratified into male *vs* female categories and comparative analysis was performed (Supplementary Fig. S6). Interestingly, in the RA cohort, the levels of five of the miRNAs, miR-29b-3p, miR-126-3p, miR-320a, let-7e-5p and let-7g-5p, were not affected by gender and had comparable levels of expression in males *vs* females. However, the levels of miR-22-3p and miR-223-3p were significantly different between males and females in RA, with miR-22-3p demonstrating a significantly higher level in males (*P* < 0.05) (Supplementary Fig. S6B) and conversely miR-223-3p exhibiting significantly higher levels in the female cohort (*P* < 0.05) (Supplementary Fig. S6C). Furthermore, when compared directly between males and females in RA alongside PsA, the levels of miR-22-3p remained significantly higher in the RA cohort compared with PsA, irrespective of gender (Supplementary Fig. S6H). However, whilst miR-223-3p expression in RA females was significantly higher than in both males and females in PsA, the expression of this miRNA in RA males was similar to the PsA cohort, failing to demonstrate a significant difference (Supplementary Fig. S6I), thus suggesting this miRNA may be a stronger RA biomarker in females compared with males. Cumulatively, the data highlight the existence of gender effects on the expression of miRNAs in RA and the importance of incorporating gender stratification into data analysis.

### Functional pathway analysis of the seven significantly different miRNAs between RA and PsA

**Figure 4:**
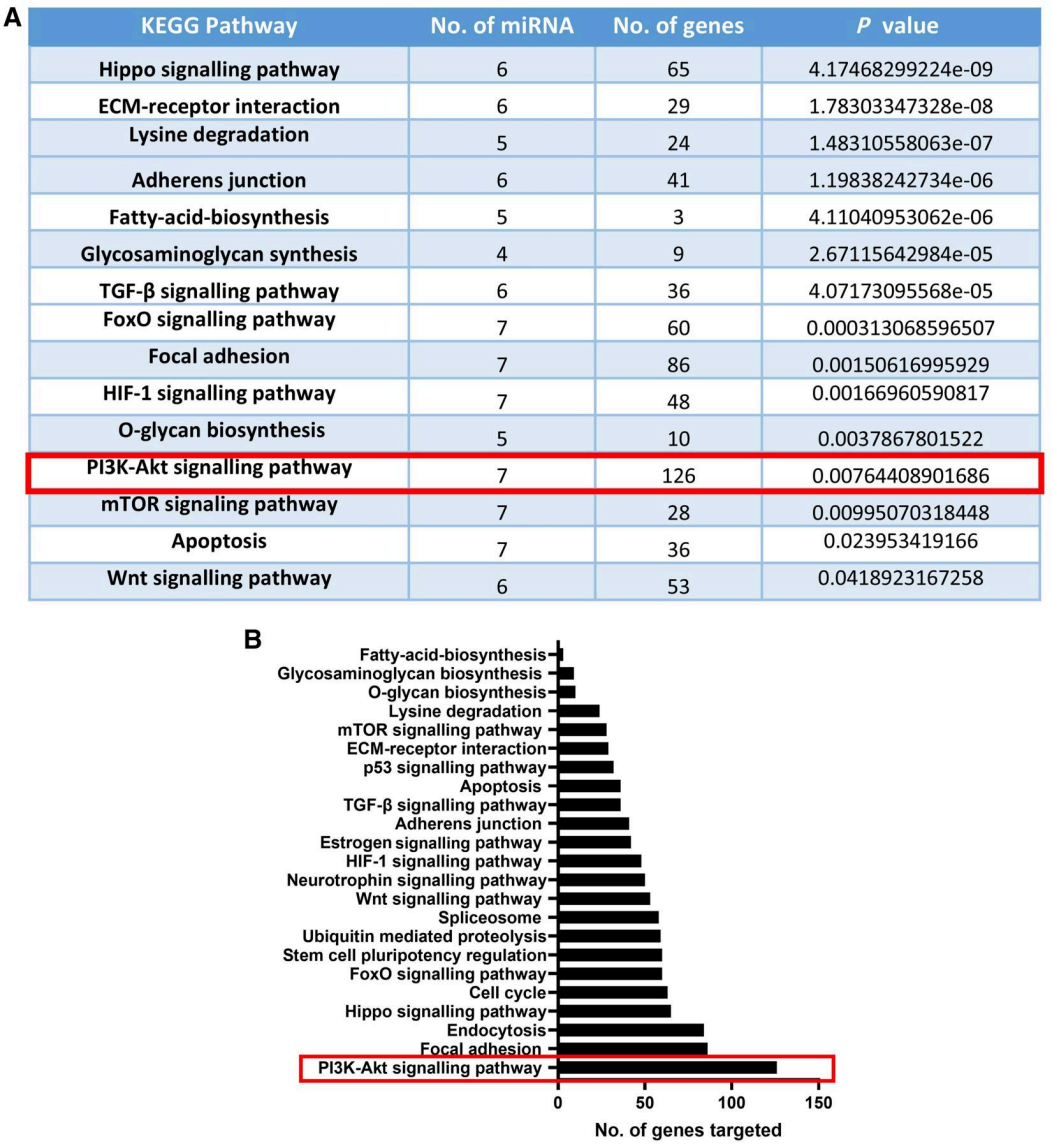
Kyoto Encyclopaedia of Genes and Genomes (KEGG) pathways enriched in miRNA target genes. The DIANA-miRPath tool was used to identify KEGG pathways enriched in genes significantly targeted by the seven differentially expressed miRNAs of interest. (**A**) Table showing relevant KEGG pathways enriched in genes experimentally proven to be targeted by the seven miRNAs of interest in decreasing order of statistical significance. (**B**) Bar graph representing the number of genes in each relevant KEGG targeted by one or more of the seven of interest

The DNA Intelligent Analysis (DIANA) miRPath tool was used to specifically recognise Kyoto Encyclopaedia of Genes and Genomes (KEGG) pathways that are enriched in genes containing predicted target sites of the identified seven miRNAs (Fig. 4A) [29]. Analysis demonstrated that the Hippo signalling pathway and the extracellular matrix (ECM) receptor interaction were the two topmost significant pathways targeted by six of the dysregulated RA serum miRNAs (*P* < 0.0001), excluding miR-22-3p (Fig. 4A). However, interestingly, the phosphoinositide-3-kinase (PI3K)–AKT signalling pathway was identified as containing the greatest number of target genes, 126 in total, regulated by all seven of the miRNAs of interest (Fig. 4A and B). Additional pathways with high significance identified included adherens junctions, the hypoxia-inducible factor-1 (HIF-1) signalling pathway, the forkhead box class O (FoxO) signalling pathway, and the mechanistic target of rapamycin (mTOR) signalling pathway (all *P* < 0.01). Furthermore, amongst the pathways being targeted by the miRNAs were certain processes directly implicated in metabolism including the anabolic reactions fatty acid biosynthesis and *O*-glycan biosynthesis, in addition to catabolic processes such as lysine degradation. However, these metabolic pathways identified were not under the control of all seven dysfunctional miRNA but by combinations of at least four of the miRNAs (Fig. 4A). The specific pathways, and the number of genes within each that are targeted by varying combinations of the seven miRNAs of interest can be found in detail in Fig. 4A and B, demonstrating the diversity and sheer complexity of pathways involved in RA *vs* PsA pathogenesis.

### Functional pathway analysis of the three significantly different miRNAs demonstrating greatest separation between RA and PsA

**Figure 5:**
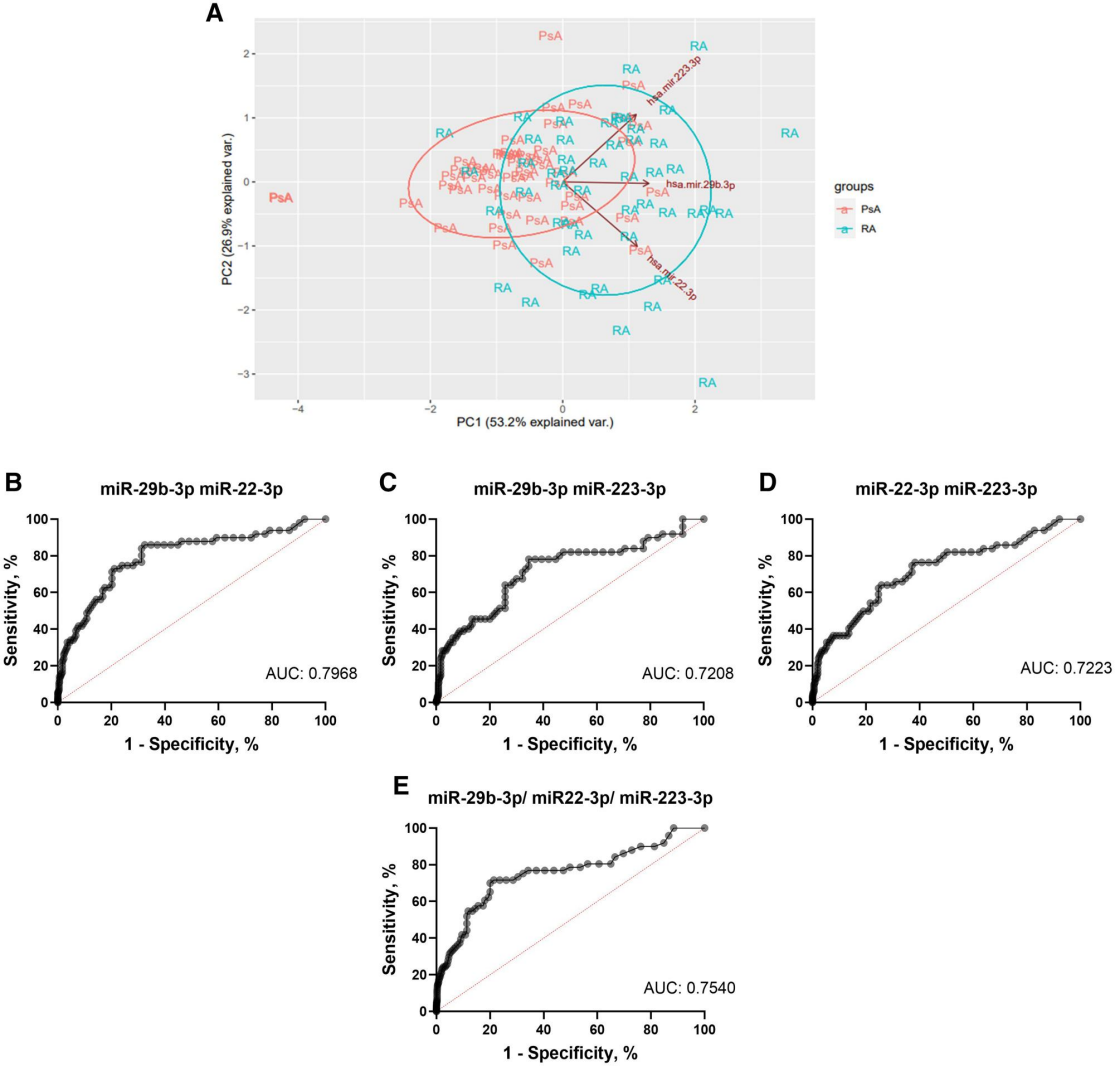
Biplot and receiver operating characteristic curve analysis of the three significant miRNAs most skewed towards RA *vs* PsA. (**A**) Principal component analysis biplots were generated under R v4.0 with package ggbiplot v0.5 with function ggbiplot. (**B–D**) The predictive value for the most differentially expressed two miRNA signatures in the serum of RA (*n* = 48) compared with PsA cohorts (*n* = 49): miR-29b-3p and miR-22-3p (**B**), miR-29b-3p and miR-223-3p (**C**), and miR-22-3p and miR-223-3p (**D**). (**E**) The predictive value for the most differentially expressed three miRNA signature in the serum of RA (*n* = 48) compared with PsA cohorts (*n* = 49): miR-29b-3p, miR-22-3p and miR-223-3p. The prediction accuracy with which each combination of miRNA can differentiate between RA and PsA is represented by the area under the curve (AUC). AUC was determined with a 95% CI

**Figure 6:**
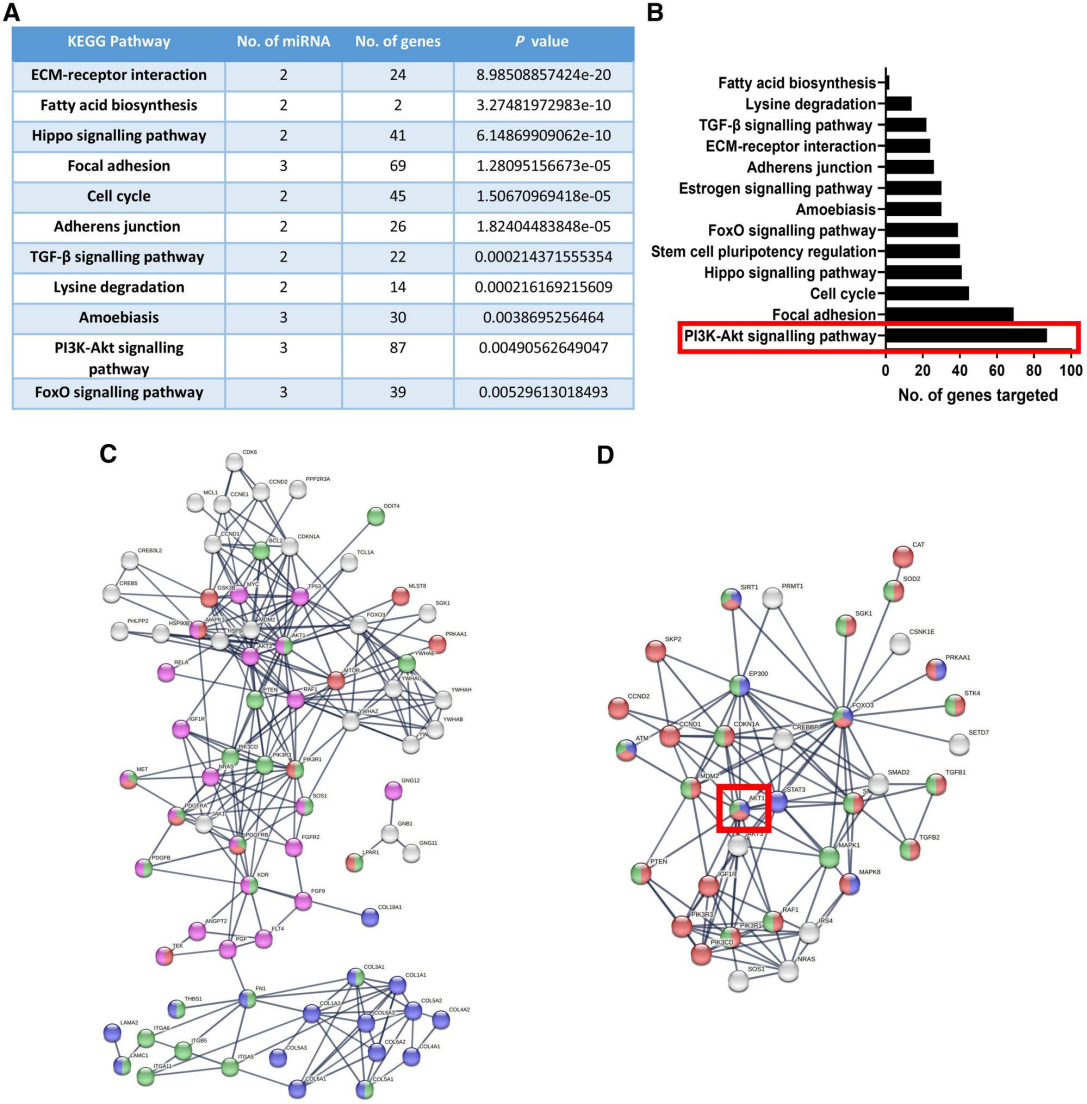
Kyoto Encyclopaedia of Genes and Genomes (KEGG) pathways enriched in miRNA target genes. DIANA-miRPath tool was used to identify KEGG pathways enriched in genes significantly targeted by the top three differentially expressed miRNAs of interest. (**A**) Table showing relevant KEGG pathways enriched in genes experimentally proven to be targeted by the three miRNAs of interest in decreasing order of statistical significance. (**B**) Bar graph representing the number of genes in each relevant KEGG pathway targeted by one or more of the three miRNAs of interest. (**C**, **D**) STRING software was used to investigate and create a visual representation of the protein–protein interactions involved in the phosphoinositide-3-kinase (PI3K)–AKT pathway (**C**) and the FoxO signalling pathway (**D**) that are targets of the top three differentially expressed miRNAs (highest confidence score = 0.9). Colour coded genes indicate protein targets involved in the following pathways in RA: (**C**) regulation of cytoskeletal organization (red nodes), extracellular matrix (ECM) organization (blue nodes), cell migration (green nodes), mitogen-activated protein kinase signalling pathway (purple nodes), and (**D**) regulation of programmed cell death (red nodes), regulation of autophagy (blue nodes), and apoptotic processes (green nodes). Red box highlights central gene implicated in the PI3K–AKT signalling pathway

While a highly significant difference in the expression of seven miRNAs between RA and PsA was identified, further biplot analysis of PCA and logplot of normalized miRNA expression demonstrated that the miRNA expression distribution was mainly associated with a dominant skew towards three specific miRNA in RA *vs* PsA: miR-29b-3p (*P* ≤ 0.0001), miR-22-3p (*P* ≤ 0.0001) and miR-223-3p (*P* ≤ 0.05) (Fig. 5A). Moreover, as shown in Fig. 5B–D, ROC curve analysis of combinations of these three specific miRNAs revealed a strong ability to distinguish between RA and PsA, with the greatest separation observed for miR-29b-3p and miR-22-3p, with an AUC of 0.7968 (Fig. 5B). The DIANA tool was then further used to analyse both the specific pathways targeted by these three significant miRNAs and the number of genes involved (Fig. 6A). The ECM-receptor interaction was the most significant pathway identified (*P* < 0.0001), with a total of 24 genes being targeted by two of the miRNAs—miR-29b-3p (20 genes) and miR-22-3p (11 genes). The PI3K–AKT signalling pathway contained the greatest number of target genes, with 87 genes being influenced by contributions from all three miRNAs of interest, more than two-thirds of the genes being regulated by all seven significantly different miRNAs. Interestingly, miR-29b-3p and miR-22-3p exerted the greatest regulation over the pathway by targeting 53 and 51 genes respectively, whilst the contribution from miR-223-3p was minimal with only six genes targeted. Additional pathways containing genes targeted by all three miRNAs included focal adhesion, amoebiasis, and the FoxO signalling pathway, all *P* < 0.01. Furthermore, anabolic pathways including fatty acid biosynthesis and catabolic pathways such as lysine degradation were also identified to be targets solely of miR-29b-3p and miR-22-3p. The specific pathways, and the number of genes within each that are targeted by varying combinations of the three miRNAs of interest can be found in detail in Fig. 6A and B.

To identify the contribution solely of these three specific miRNAs in targeting the PI3K–AKT signalling pathway, the STRING software tool was then used to visually analyse the complex protein network involving the 87 genes (Fig. 6C). Among the targets of interest were several processes highly associated with the invasive and inflammatory capacity of cells that perpetuate damage within the inflamed synovium including regulation of cytoskeletal organization (Fig. 6C, red), ECM organization (Fig. 6C, blue), cell migration (Fig. 6C, green), and the mitogen-activated protein kinase signalling pathway (Fig. 6C, purple). The specific proteins involved in mediating these various processes included growth factors such as fibroblast growth factor-9, placental growth factor and platelet-derived growth factor-β, the adhesive glycoprotein thrombospondin-1, structural proteins including fibronectin-1 and collagen family members, in addition to several members of the AKT/mTOR signalling pathway such as MYC, AKT1, AKT3 and mTOR itself. These results suggest the central role these three miRNAs assume in facilitating structural alterations and migratory processes within the inflamed joint and thus their potential benefit as therapeutic targets. Moreover, due to the involvement of all three miRNA in its regulation and the high number of gene targets (39), the FoxO pathway signalling interactions were also examined using STRING analysis (Fig. 6D). Among the primary targets of interest in this pathway were several processes related to cell survival and death including the regulation of programmed cell death (Fig. 6D, red nodes), regulation of autophagy (Fig. 6D, blue nodes), and apoptotic processes (Fig. 6D, green nodes). Interestingly, the *AKT1* gene also assumed a central role in this network where it demonstrated a function in the mediation of all three aforementioned processes, highlighting the potential importance of AKT1 in mediating not solely the PI3K–AKT pathway but additionally its interconnection with FoxO-mediated signalling. Cumulatively, these results would imply that these three miRNAs of interest assume an integral role in regulating not only migratory but additionally apoptotic and inflammatory processes within the inflamed joint and thus may prove highly valuable in ameliorating RA pathogenesis if targeted therapeutically.

## Discussion

The identification of biomarkers that provide a non-invasive and reliable method of enabling early diagnosis of autoimmune diseases including RA constitutes an area of intense research focus as it has been well-established that earlier diagnosis precedes superior outcomes [20]. Unfortunately, despite only being present in 70% of RA patients, ACPA positivity remains the only validated biomarker currently utilized diagnostically in clinical settings [20, 21]. Consequently, investigation of the biomarker potential of serum miRNAs and their role in the initial development and progression of RA is of particular importance [6]. In this present study, 19 serum miRNAs related to immune dysfunction were identified, demonstrating significant differences between RA and PsA cohorts. However, further interrogation revealed that seven of these miRNAs could function as potential biomarkers assisting in the early discrimination of RA from PsA, with all seven exhibiting good sensitivity and specificity for RA *vs* PsA. These seven miRNAs were miR-126-3p, miR-29b-3p, miR-22-3p, miR-223-3p, miR-320a, let-7e-5p and let-7g-5p, all of which were significantly increased in RA serum compared with PsA serum, and importantly, compared with HC. Further biplot investigation determined that three of these miRNAs in particular were skewed predominantly towards RA compared with PsA—miR-22-3p, miR-29b-3p and miR-223-3p. Downstream analysis of their potential gene targets resulted in the identification of several key pathways implicated in processes including angiogenesis, immune dysfunction and cell death, all of which are fundamental pathogenic events driving RA synovial joint destruction. Cumulatively, the results suggest that these three serum miRNAs may prove highly valuable, not solely as therapeutic targets, but as diagnostic markers enabling prompt discrimination of RA from PsA pathology.

Extensive literature has shown that miRNAs play key roles in many human diseases, particularly autoimmune disorders [21]. These findings support earlier studies demonstrating altered circulating miRNA levels, reinforcing aberrant miRNA expression in autoimmune pathologies [4, 9]. Indeed, previous studies have illustrated the various roles different miRNAs assume in RA pathogenesis including miR-155 and miR-146, in addition to miR-31-5p, miR-126-3p, miR-221-3p, miR-24-3p and miR-339-5p, amongst others, all of which have been shown to govern the functioning of innate and adaptive immune cell populations, proinflammatory cytokine release and nuclear factor-κB pathway transcriptional activation [6, 9, 19]. Furthermore, although less extensive, recent work in PsA has highlighted panels of specific miRNAs that demonstrate abnormal serum expression levels in patients compared with HC including miR-21-5p, miR-23a, miR-26a-5p, miR-125a, miR-130a-3p, miR-146a-5p, miR-151-5p and miR-221-3p4 [12, 13]. However, this is the first study to specifically determine whether expression levels of this specific panel of serum miRNA could prove efficacious in discriminating RA and PsA patients thus providing novel insights.

Initial analysis identified seven miRNAs with significantly different expression between RA and PsA; however, expanded biplot analysis highlighted three miRNAs that contributed most to the separation between the two diseases, suggesting their potential as independent biomarkers. Gender stratification revealed that two of these RA-associated miRNAs differed significantly by gender within the RA cohort, emphasizing the importance of sex-based analysis. Functionally, miR-22-3p, miR-29b-3p and miR-223-3p have been implicated in inflammatory diseases and cancer biology [30–34]. miR-22-3p upregulation has been shown to suppress cell migration and epithelial–mesenchymal transition in cancer [34], and although its role in RA is less defined, elevated serum levels, consistent with our findings, have been linked to RA development in ACPA-positive individuals [35]. Furthermore, miR-22-3p has been proposed as a diagnostic biomarker for RA, with higher serum levels observed in RA compared with HC [31, 36].

Similarly, miR-223-3p has been shown to be increased in RA synovial fluid compared with HC and OA comparators, in addition to demonstrating altered expression in early RA, suggesting involvement in RA pathogenesis [37, 38]. Moreover, in line with our findings, previous analysis revealed levels of miR-223-3p were significantly higher in serum and synovial tissue of RA individuals compared with HC, and when considered alongside miR-146a could improve predictive diagnostic accuracy [39]. The functional implications of this miRNA are extremely important in the context of inflammation with several studies highlighting the strong association of miR-223-3p with NLRP3 inflammasome regulation, with targeting of this interaction shown to induce apoptosis of pathogenic RA fibroblast-like synoviocytes (FLS) [40, 41]. Interestingly, although the role of miR-29b-3p in cancer is significantly more established than its autoimmune function, studies have shown miR-29b-3p to be critical in regulating angiogenesis in the tumour microenvironment, through AKT3–vascular endothelial growth factor (VEGF) signalling [42]. In the context of RA and PsA, elevated expression of VEGF is integral to the characteristic extracellular migration and subsequent pathological angiogenesis, indicating a potential role for this miRNA in this phenomenon [43].

Functional pathway analysis of the seven significantly different miRNAs most strongly associated with RA compared with PsA indicated that the PI3K–AKT pathway is a key target, with the greatest number of associated target genes. Interestingly, these results were mirrored in analysis of the top three significantly different miRNAs skewed towards RA, thus not only highlighting the pivotal role of the PI3K–AKT pathway in RA disease propagation, but also the valuable biomarker and therapeutic potential of these three miRNAs. The PI3K–AKT pathway is involved in a diverse range of cellular processes including cell growth, proliferation, regulation of metabolism and angiogenesis, and the release of multiple proinflammatory cytokines that perpetuate or sustain inflammation [44]. Indeed, previous success has been achieved in treating immune-mediated arthritis by targeting this pathway with PI3K small molecule inhibitors [44]. However, of particular significance is the network of genes within this pathway identified to be potential targets of the RA miRNAs, including metabolic/invasive factors, *mTOR, SIRT1, RAC-1, FN-1, ITGA5* and *AKT3*, in addition to several collagens, all implicated in cytoskeletal rearrangement and promotion of invasion [45]. The identification of these genes as potential targets for alleviating the pro-migratory capacity of cells within the synovium is supported by previous studies showing that direct blockade of the mTOR pathway, in which AKT proteins serve as upstream regulators, can have suppressive effects on invasive RA-FLS [46]. Furthermore, the FoxO signalling pathway was targeted by all three miRNAs of interest, with this cascade previously shown to assume a central role in angiogenic tip cell selection though its governance over VEGF-responsive tip cell-enriched genes [47]. In the context of RA, it has been established that FoxO family members are key in the modulation of cell proliferation and survival, with studies showing that reduced FoxO1 expression promotes FLS survival, and additionally miR-155 directly targets FoxO3a in FLS resulting in heightened proinflammatory and proliferative capacity [48, 49]. Indeed, key potential gene targets of the three miRNAs were identified as being central to programmed cell death and apoptotic processes including *SIRT1*, *PTEN*, *RAF1* and *ATM*, in addition to *AKT1*, reinforcing the valuable nature of potentially targeting this pathway to restore normal homeostatic states in pathogenic RA cells. Previous literature has highlighted a clear interconnected network between PI3K–AKT and FoxO signalling, with an eloquent study identifying a reduction in pathogenic FLS apoptosis induced through AKT1 inhibition of FoxO1 [50]. This interaction may be of particular interest in the context of RA therapeutic intervention as it suggests that targeting the three miRNAs could exert beneficial effects on not one but a myriad of pathways driving RA pathogenesis.

Collectively, these findings highlight the therapeutic potential of targeting miRNAs to modulate multiple pathways of synovial inflammation and joint damage. Furthermore, the identified circulating miRNA signature shows strong potential for distinguishing RA from PsA, while importantly revealing candidate therapeutic targets and disease-specific pathways.

## Supplementary Material

keag246_Supplementary_Data

## Data Availability

The data underlying this article will be shared on reasonable request to the corresponding author.
