# Supplementary material for: Circulatory microRNA signature distinguishing rheumatoid arthritis and psoriatic arthritis
Source: Rheumatology (Oxford). 2026 May 8;65(6):keag246. doi: 10.1093/rheumatology/keag246 (PMC13275133; doi:10.1093/rheumatology/keag246)
Supplement: keag246_Supplementary_Data [file keag246_supplementary_data.zip › rhe-25-3198-File009.pdf]

Supplementary

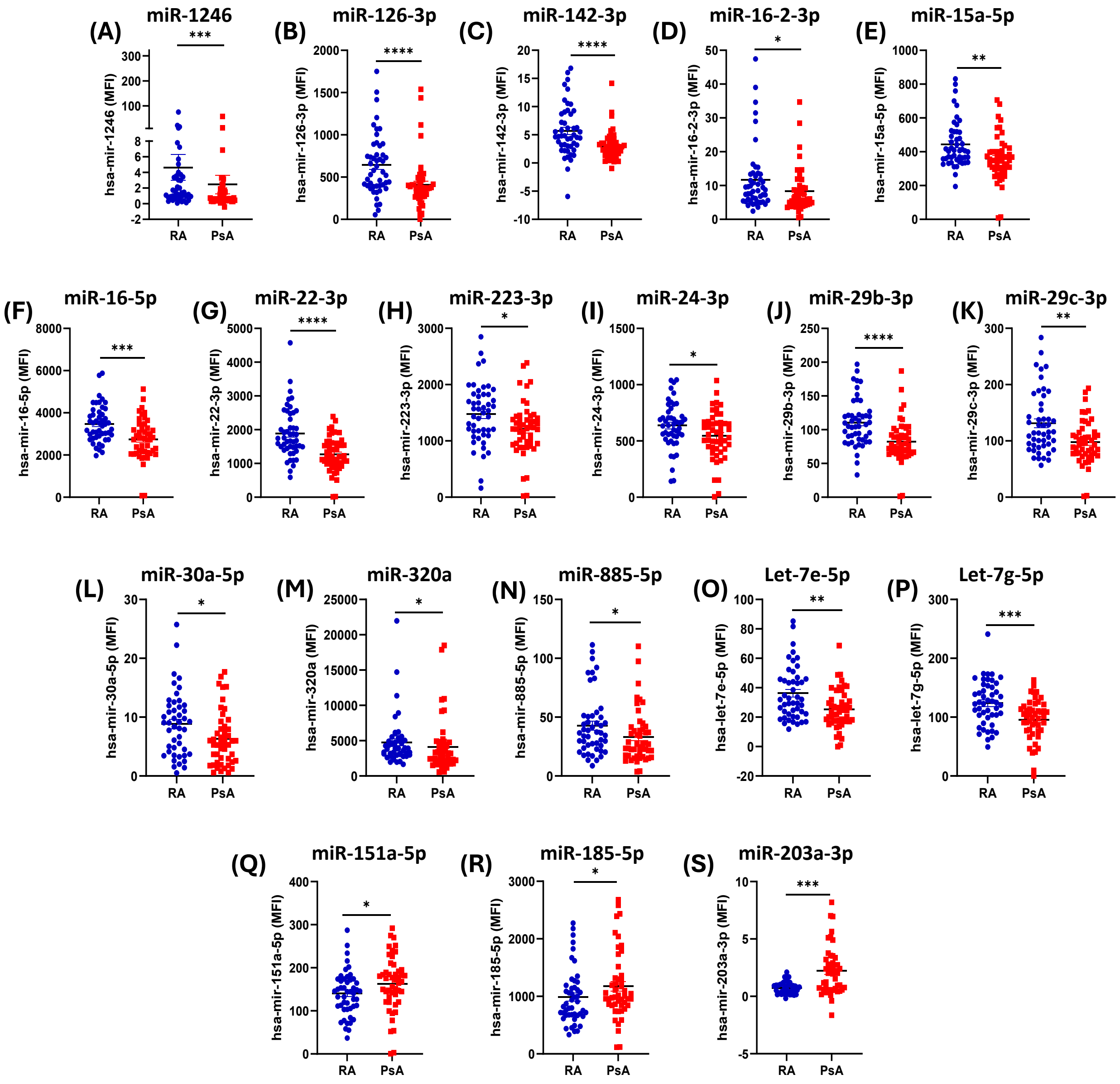

Supplementary Figure S1. Identification of nineteen differentially expressed circulating miRNA between RA and PsA. Serum was isolated from the blood of RA (n=48) and PsA (n=49) patients and a panel of 68 miRNA was analysed using the Multiplex Circulating miRNA Assay. **(A-S)** Representative dot plots demonstrating the difference in expression MFI of the nineteen differentially expressed miRNAs of interest between the RA and PsA patient cohorts. All data is represented as Mean +/- SEM. Statistical analysis was performed using the non-parametric, Mann-Whitney *U* Test with statistical significance defined by *p≤0.05, **p≤0.01, ***p≤0.001, and ****p≤0.0001.

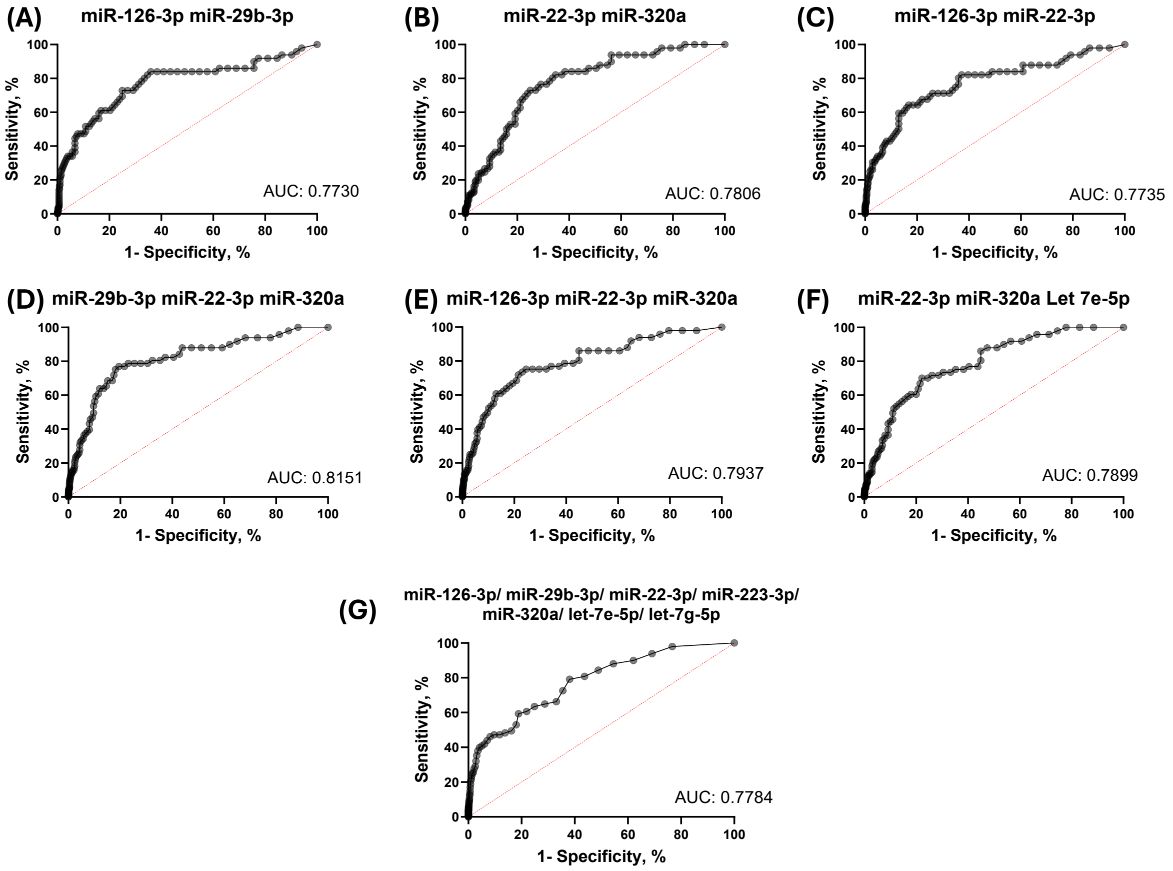

Supplementary Figure S2. ROC curve analysis based on the expression levels of multivariant serum miRNA. The diagnostic value for multivariant serum miRNA for two-miRNA signature; **(A)** miR-126-3p and miR-29b-3p; **(B)** miR-22-3p and miR-320a; and **(C)** miR-126-3p and miR-22-3p, three-miRNA signature; **(D)** miR-29b-3p, miR-22-3p, and miR-320a; **(E)** miR-126-3p, miR-22-3p, and miR-320a; and **(F)** miR-22-3p, miR-320a, and let-7e-5p, with the strongest individual AUC values. **(G)** The predictive value of the seven most differentially expressed miRNA signature. The prediction accuracy with which each combination of miRNA can differentiate between RA and PsA is represented by the AUC. AUC was determined with a 95% confidence interval.

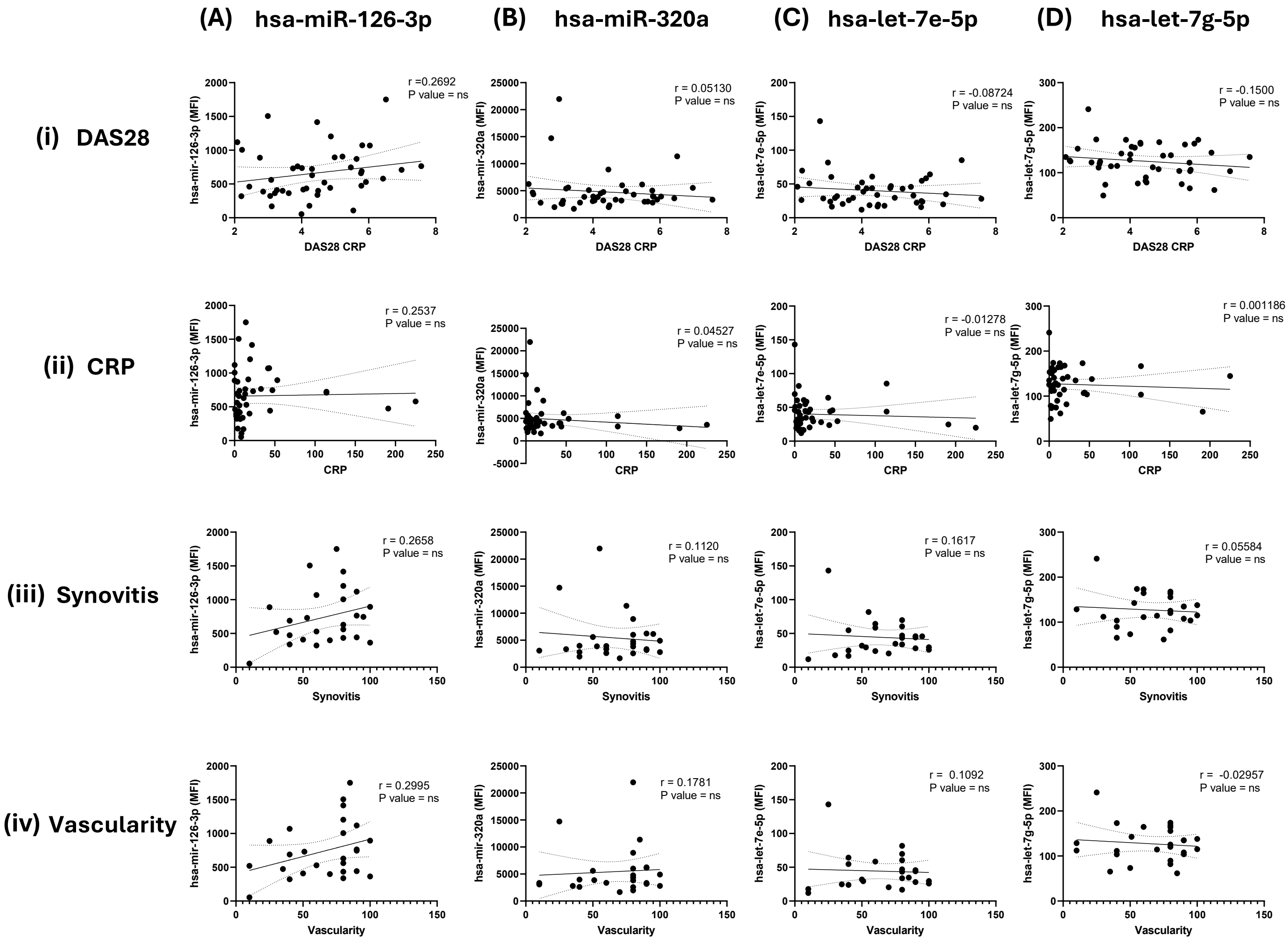

Supplementary Figure S3. No correlation between serum miRNA and clinical parameters in RA and PsA cohorts. (A-D) Spearman pair-wise correlation analysis of miRNA expression and **(i)** DAS28-CRP, **(ii)** CRP, **(iii)** synovitis, and **(iv)** vascularity in RA and PsA cohorts (n=24-43). Simple linear regression model was used with unknown standards interpolated from the curve and the data presented with the 95% confidence bands of the best fit.

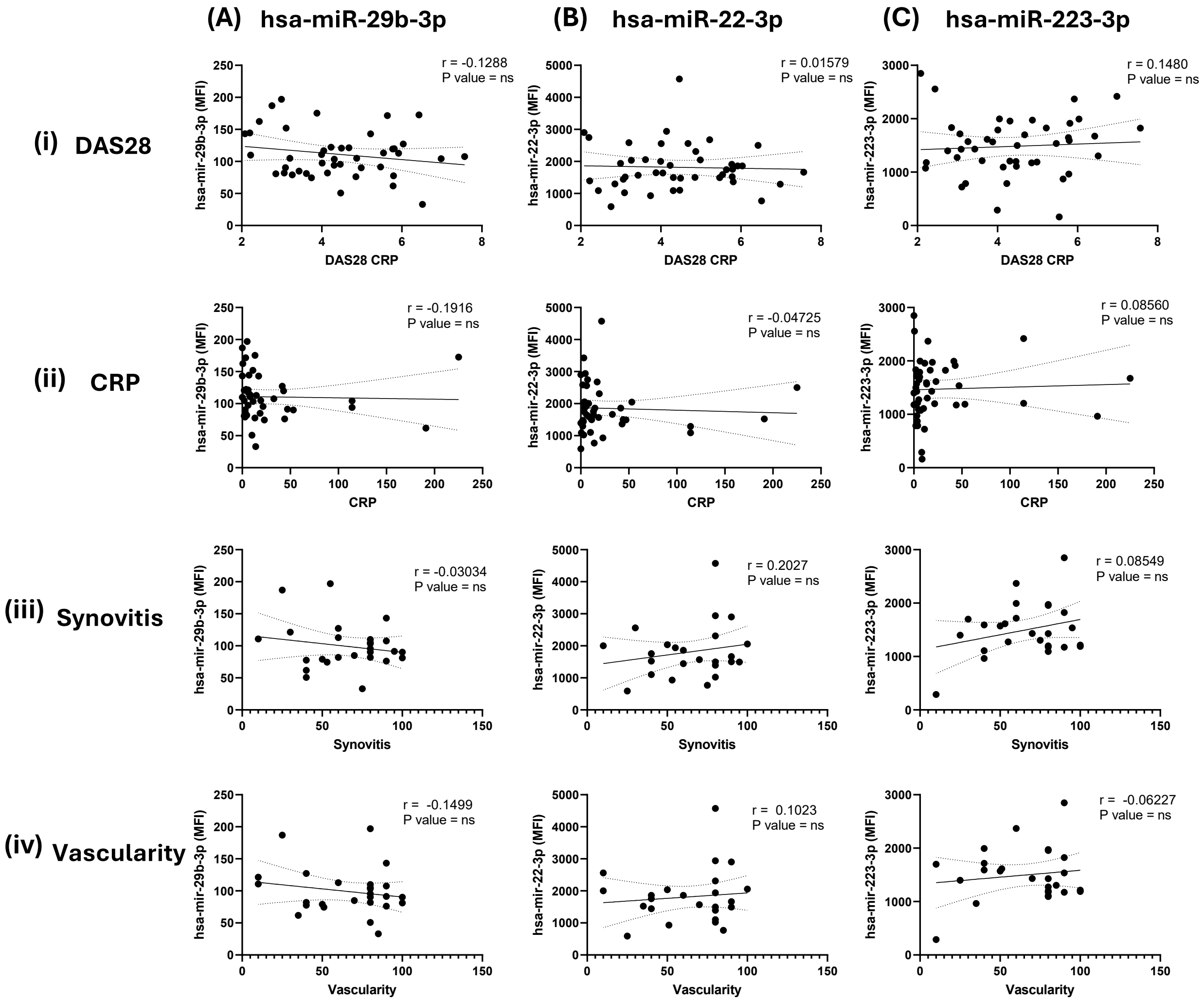

Supplementary Figure S4. No correlation between serum miRNA and clinical parameters in RA and PsA cohorts. (A-C) Spearman pair-wise correlation analysis of miRNA expression and **(i)** DAS28-CRP, **(ii)** CRP, **(iii)** synovitis, and **(iv)** vascularity in RA and PsA cohorts (n=24-43). Simple linear regression model was used with unknown standards interpolated from the curve and the data presented with the 95% confidence bands of the best fit.

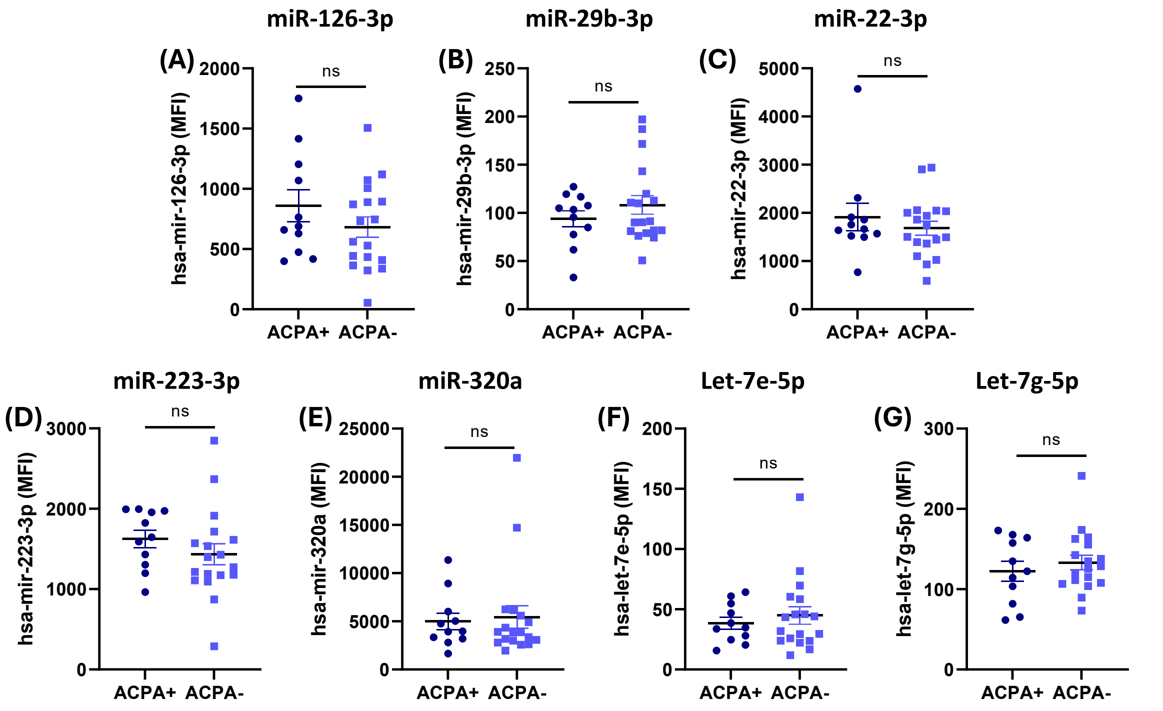

Supplementary Figure S5. ACPA status did not alter levels of the seven significantly increased serum miRNA in RA donors. (A-G) Dot plot representations showing MFI of the relative expression of seven miRNA of interest isolated from the serum of a subset of RA donors; RA ACPA^{+} (n=11) versus RA ACPA^{-} (n=18), analysed and amplified using the Multiplex Circulating miRNA Assay. All data is represented as Mean +/- SEM.

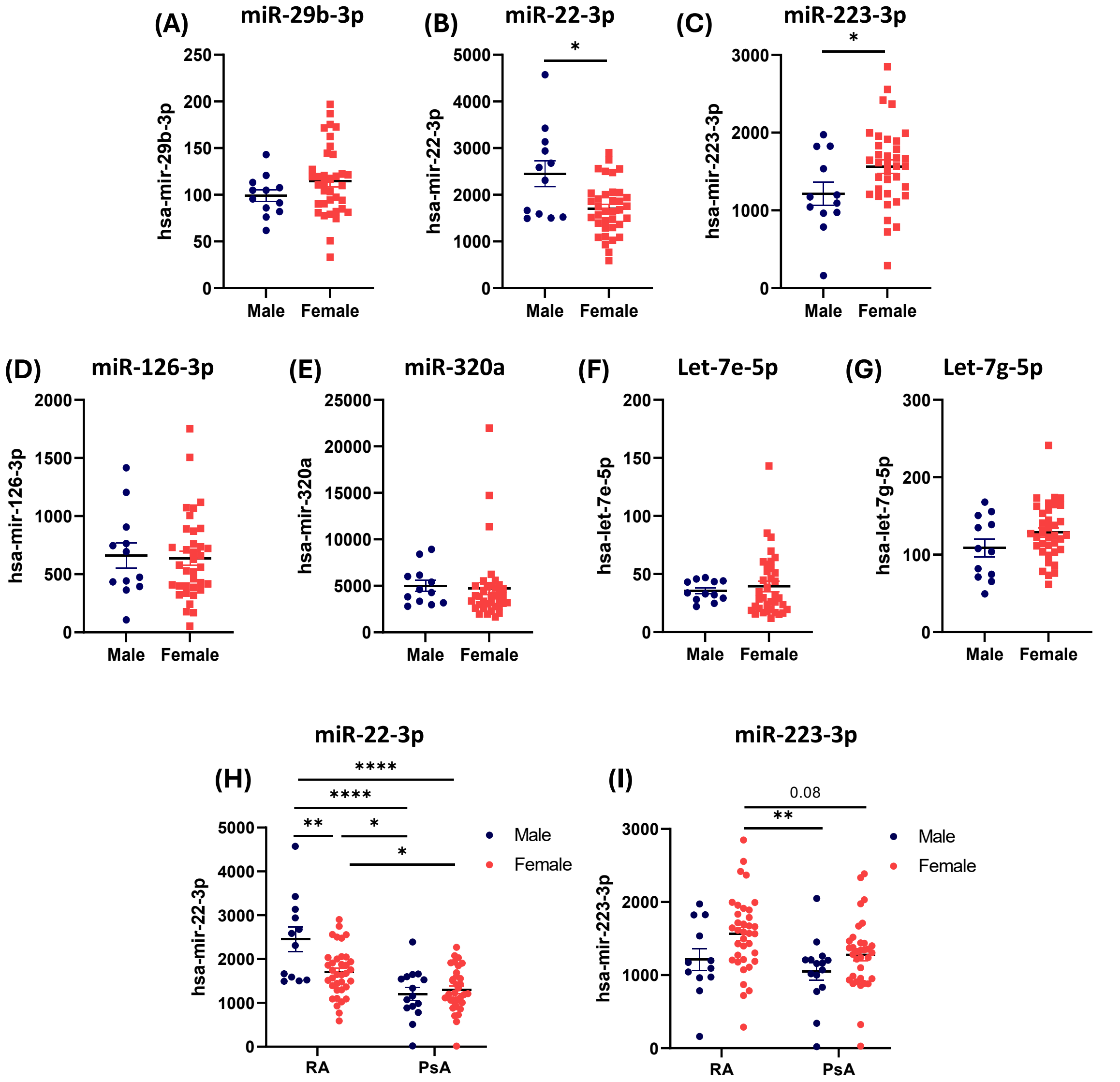

Supplementary Figure S6. Differences in the expression of specific miRNA between males and females in the RA cohort. (A-G) Dot plot representations showing MFI of the relative expression of the seven miRNA of interest when compared directly between males (n=12) and females (n=36) in the RA cohort. **(H-I)** Dot plot representations showing MFI of the relative expression of miR-22-3p and miR-223-3p when compared directly between RA males (n=12), RA females (n=36), and the PsA cohort (male = 15, female = 34). All data is represented as Mean +/- SEM. Statistical analysis was performed using the non-parametric, Mann-Whitney *U* Test or Kruskal-Wallis Test with statistical significance defined by *p≤0.05, **p≤0.01, ***p≤0.001, ****p≤0.0001.

Differentially expressed miRNA	P Value
**Higher expression in RA**	
**miR-126-3p**	P<0.0001
**miR-22-3p**	P<0.0001
**miR-29b-3p**	P<0.0001
**miR-142-3p**	P<0.0001
**miR-1246**	P<0.001
**miR-16-5p**	P<0.001
**Let-7g-5p**	P<0.01
**Let-7e-5p**	P<0.01
**miR-15-5p**	P<0.01
**miR-29c-3p**	P<0.01
**miR-30a-5p**	P<0.05
**miR-885-5p**	P<0.05
**miR-16-2-3p**	P<0.05
**miR-24-3p**	P<0.05
**miR-223-3p**	P<0.05
**miR-320a**	P<0.05
**Higher expression in PsA**	
**miR-203a-3p**	P<0.001
**miR-185-5p**	P<0.05
**miR-151a-5p**	P<0.05

Supplementary Table S1. Table depicting the top nineteen differentially expressed serum miRNA between RA (n=48) and PsA (n=49) cohorts and their corresponding P value. Statistical analysis was performed using the non-parametric, Mann-Whitney *U* Test with statistical significance defined by *p≤0.05, **p≤0.01, ***p≤0.001, ****p≤0.0001.
