# Supplementary material for: Circulatory microRNA signature distinguishing rheumatoid arthritis and psoriatic arthritis
Source: Rheumatology (Oxford). 2026 May 8;65(6):keag246. doi: 10.1093/rheumatology/keag246 (PMC13275133; doi:10.1093/rheumatology/keag246)
Supplement: keag246_Supplementary_Data [file keag246_supplementary_data.zip › 02-Jun-2026_074005_rhe-25-3198-File008.docx]

## **Supplementary Data S1**

## **Materials & Methods**

### **Patient Recruitment**

Patients with inflammatory arthritis (IA) were previously recruited from the outpatient clinic at the Department of Rheumatology, St Vincent’s University Hospital. Patients with rheumatoid arthritis (RA) (n=48) and psoriatic arthritis (PsA) (n=49) fulfilled the criteria established by the American College of Rheumatology (ACR)/ European Alliance of Associations for Rheumatology  (EULAR) and the Classification Criteria for Psoriatic Arthritis (CASPAR) respectively^1,2^. Patient demographics and baseline clinical and laboratory assessments including c-reactive protein (CRP), swollen joint count (SJC), tender joint count (TJC), the visual analogue scale (VAS), synovitis, and vascularity are summarized in *Table 4.1*. Twenty healthy controls (HC), all of which were negative for autoimmune disease, were recruited and included in the study for comparative purposes.

            All patients and controls provided fully informed written consent before commencing the study. Ethical approval was granted by St. Vincent’s University Hospital Medical Research and Ethics Committee, and all research was performed in accordance with the Declaration of Helsinki.

| Patient/ Clinical Info. | RA (n=48) | PsA (n=49) | HC |
| --- | --- | --- | --- |
| Age median (range) | 58 (27-83) | 46 (34-58) | 38 (27-49) |
| Gender (F:M) | 36:12 | 34:15 | 13:7 |
| DAS28 CRP +/-SEM | 4.42 +/- 0.21 | 3.70 +/- 0.16 | NA |
| CRP (mg/dl) +/-SEM | 26.22 +/- 7.02 | 11.2 +/- 2.78 | NA |
| SJC +/SEM | 4.98 +/- 0.69 | 2.73 +/- 0.50 | NA |
| TJC +/-SEM | 5.93 +/-0.99 | 3.4 +/- 0.59 | NA |
| VAS +/-SEM | 61.45 +/- 4.47 | 59.43 +/- 1.97 | NA |
| Synovitis +/-SEM | 65.89 +/- 4.8 | 70.03 +/- 3.26 | NA |
| Vascularity +/-SEM | 66 +/- 5.27 | 66.45 +/- 3.57 | NA |

Patient demographics. Summary of RA (n=48) and PsA (n=49) patient demographics and clinical parameters, as well as age and gender for the HC comparators (n=20). Clinical parameters are presented as Mean +/- SEM. DAS: Disease Activity Score, CRP: C-Reactive Protein, SJC: Swollen Joint Count, TJC: Tender Joint Count, VAS: Visual Analogue Scale, SEM: Standard Error of Mean.

### **Serum miRNA Expression Analysis**

Blood was obtained from three cohorts; serum was isolated and subsequently transferred to RNase and DNase free tubes. The FirePlex Circulating 68 miRNA Immunology Fixed Panel was performed as a service by Abcam, Cambridge, UK. This specific multiplex panel used focused on miRNA associated with immune and stromal cell dysfunction characteristic of autoimmune diseases. While there are many different methods of miRNA analysis, the FirePlex method was utilised as a high-throughput platform as this method reliably quantifies miRNA directly from biofluids with minimal sample preparation, therefore reducing the miRNA isolation-induced variability.

             Targeted hybridization of serum miRNA to miRNA probes involved the incubation of 20 μl of patient serum with 35 μl of FirePlex hydrogel particles in addition to incubation with 25 μl of hybridization buffer with gentle agitation at 37°C for 1 h. Using rinsing Buffer A, samples were rinsed twice before 75 μl of labelling mix was added. This solution was then incubated at room temperature (RT) for 1 h with gentle agitation. Following rinsing with Buffer B (x 2) and Buffer A (x 1), 110 μl of RNase-free water was added to the solution and was incubated at 55°C for 30 min. Ligated miRNAs were then eluted and amplified using single-step universal primer real-time amplification which consisted of 27 cycles of polymerase chain reaction (PCR) amplification followed by 6 cycles of asymmetric amplification. Following this, the amplified products were rehybridized with FirePlex particles in 75 μl of reporting buffer and incubated at RT for 15 min whilst gently agitated. To enable fluorescent detection of the particle-bound miRNA, 175 μl of run buffer was then added prior to reading on the EMID Millipore Guava 8HT flow cytometer. Background signals were corrected for using internal positive, negative, and blank controls. Further interpretation and analysis of assay results was performed using the FirePlex Analysis Workbench software.

### **Bioinformatic Analysis**

miRNA expression data were analysed in R v4.0 following scaling. Principle component analysis (PCA)-biplots were generated under R v4.0 with package ggbiplot v0.5 with function ggbiplot. The DNA Intelligent Analysis (DIANA)-miRPath online software suite was used to perform miRNA pathway analysis. This was performed to identify downstream Kyoto Encyclopaedia of Genes and Genomes (KEGG) pathways and genes potentially altered by the dysregulated serum miRNA being evaluated in this study^3^. STRING software was subsequently used to generate visual representations of the protein-protein interactions of the genes involved in the pathways targeted by the dysregulated miRNAs^4^.

### **Statistical Analysis**

All statistical analysis was performed using GraphPad Prism 9 software (GraphPad Software Inc., California, USA). The nonparametric, Mann-Whitney *U* test was used to determine significant differences between RA and PsA samples whilst the one-way ANOVA and Kruskal-Wallis test was used to determine significant differences between RA, PsA, and HC. All data was presented as Mean +/- SEM with statistical significance defined by *p≤0.05, ** p≤0.01, *** p≤0.001, **** p≤0.0001. Receiver operating characteristic (ROC) curves were performed using Prism 9 Software to establish the ability of a specific miRNA or miRNA combination to discriminate between RA and PsA using the area under the curve (AUC), determined using a 95% confidence interval (CI). Spearman rank correlation coefficient was used to evaluate the association between miRNA levels and clinical variables.

**Bibliography**

1. Aletaha D, Neogi T, Silman AJ, et al. 2010 Rheumatoid arthritis classification criteria: an American College of Rheumatology/European League Against Rheumatism collaborative initiative. Arthritis Rheum 2010;62:2569-81.

2. Taylor W, Gladman D, Helliwell P, Marchesoni A, Mease P, Mielants H. Classification criteria for psoriatic arthritis: development of new criteria from a large international study. Arthritis Rheum 2006;54:2665-73.

3. Vlachos IS, Zagganas K, Paraskevopoulou MD, et al. DIANA-miRPath v3.0: deciphering microRNA function with experimental support. Nucleic Acids Res 2015;43:W460-6.

4. Szklarczyk D, Gable AL, Lyon D, et al. STRING v11: protein-protein association networks with increased coverage, supporting functional discovery in genome-wide experimental datasets. Nucleic Acids Res 2019;47:D607-d13.
